# Desmoid tumor of the supraclavicular region: a case report

**DOI:** 10.4076/1757-1626-2-7222

**Published:** 2009-06-22

**Authors:** Ilias Koukoutsis, Apostolos Pappas, George Karanikas, Katerina Kotzadimitriou, John Chrysikos, Styliani Tzika, Nikolaos Koronakis, George Karavitis, Emmanuel Lagoudianakis, Andreas Manouras

**Affiliations:** 1 2nd Department of Surgery, 417 NIMTS (Military Veterans' Fund Hospital), Monis Petraki 10, 11521, Athens, Greece; 2 1st Department of Propaedeutic Surgery, Hippocrateion Hospital, Athens Medical School, Q. Sophia 114, 11527, Athens, Greece

## Abstract

Desmoid tumors are rare, benign fibroblastic tumors that are locally infiltrative and can cause extensive morbidity by destruction of adjacent vital structures. Due to the rarity of these tumors, evidence regarding optimal treatment protocols is drawn from case reports and single-arm series with small patient numbers. We report a case of a patient with a desmoid tumor of the left supraclavicular region that was diagnosed and treated in our department and a review of the current literature.

## Introduction

The fibromatoses or desmoid tumors are a group of rare disorders arising from the connective tissue of the muscle, the overlying fascia or aponeurosis [1]. Although they are regarded as benign lesions, desmoid tumors are locally infiltrative and can cause extensive morbidity by destruction of adjacent vital structures and organs [2]. Incidence is estimated at 2.4 to 4.5 cases per million persons annually, while most cases appear to be intra-abdominal with only 7-15% of cases involving the head and neck [3]. The rarity of these tumors has hindered the progress of the studies on this disease and thus, to date, the optimal treatment protocol for these patients remains unclear [4]. We report a case of a patient with fibromatosis of the left supraclavicular region that was diagnosed and treated in our department.

## Case presentation

**Figure 1:**
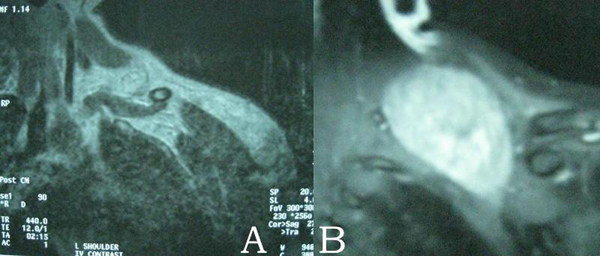
**Magnetic resonance imaging of the neck shows on T1 (A) and T2 (B) sequencing a well-circumcised mass with increased signal intensity in the posterior cervical triangle, extending to the thoracic cavity**.

**Figure 2:**
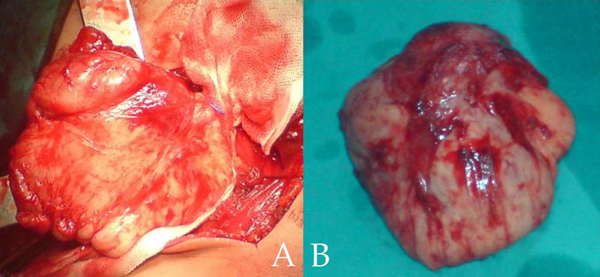
**(A) Gross total resection of the tumor**. **(B)** Macroscopic appearance of the excised mass.

A 21-year-old Caucasian Greek male with no past medical history, presented with an enlargement in his left supraclavicular region associated with numbness in his left arm during physical activity. Upon physical examination, there was a smooth, firm, non-tender supraclavicular mass and no palpable lymph nodes. Neurological examination of the upper extremities did not show any sensory or motor deficits. Magnetic resonance imaging (MRI) of the neck revealed an enhancing mass in the posterior cervical triangle, extending to the thoracic cavity (Figure 1). Fine-needle aspiration biopsy was performed but the results were inconclusive. The patient underwent surgery, during which the tumor was located posterior to the brachial plexus and was extending into the thoracic cavity behind the parietal pleural. Gross total resection of the tumor was obtained, leaving unharmed the neural elements as well as the large vessels of this area. The excised tumor, measuring 10 cm × 8 cm × 4.5 cm, had well defined smooth, round borders and was relatively firm (Figure 2). Histological examination of the mass revealed the presence of elongated spindle-shaped cells, surrounded by abundant collagen, features consistent with fibromatosis. Immunostaining was positive for desmin, vimentin, non-specfic enolase and b-catenin and negative for acinin and S-100. The patient had an uneventful postoperative course and was discharged on the 4^th^ postoperative day. During his follow-up MRI study, 6 months after his operation, no evidence of tumor recurrence were detected.

## Discussion

Desmoid tumors are rare fibroblastic tumors that are characterized by the presence of proliferating normal-appearing fibroblastic cells in an abundant collagen stroma. There is a female predominance while the highest incidence arises among patients between the ages of 15 to 60 years old [5]. Desmoids arise sporadically, nevertheless in an incidence ranging from 3% to 35% these tumors can occur in patients with familial adenomatous polyposis [6,7].

Desmoid fibromatosis may occur at abdominal, intra-abdominal and extraabdominal locations. Extraabdominal fibromatosis most frequently occurs in the limbs, followed by the head and neck region [8]. Although these tumors are benign their clinical behavior is unpredictable. Rock et al, in a series of 194 patients with extra-abdominal desmoid tumors, report a 68% recurrence rate at an average of 1.4 years after the first treatment [9]. Such an aggressive behavior combined with the presence of many vital structures in a confined area in the head and neck, consists of a major therapeutic challenge.

At present the management of desmoid tumors includes the use of surgery, radiation therapy (RT) as well as cytotoxic and noncytotoxic chemotherapy. Given the benign nature of these tumors, treatment strategies aim at achieving local control while preserving proper function and providing with an adequate cosmetic result [4]. When medically and technically feasible, complete resection of the tumor with negative microscopic margins is the first-line treatment [10]. Nevertheless, it must be noted that the importance of positive margin status to local recurrence rates, is still a matter of debate. In contrast to other published series [2,10], a study by Gronchi et al showed that presence of microscopic disease did not affect long-term disease-free survival in patients with primary presentation of extra-abdominal desmoid tumors [11]. Primary RT is an appropriate alternative for patients who are not good surgical candidates [12]. On the other hand the use of postoperative RT is unclear. After taking under consideration the controversy regarding the significance of positive resection margins and the potential for late radiation toxicity, particularly in younger patients, some researchers advocate that RT should not be pursued in patients with primary disease and either negative or positive surgical margins, but only in cases with gross residual disease [13,14]. In agreement with this approach our patient did not receive adjuvant RT. Systemic therapy includes a variety of noncytotoxic (tamoxifen, testolactone and the nonsteroidal anti-inflammatory drugs) and cytotoxic agents (methotrexate, vinblastine and doxorubicin). Possible candidates for systemic therapy include patients with Gardner's syndrome and unresectable or recurrent desmoid tumors, involving the mesentery [15]. It must be emphasized that, to date, the evidence regarding the efficacy of these agents are drawn from case reports and single-arm series with small patient numbers [16].

In summary, desmoid tumors are rare and benign neoplasms with a unique biologic behavior. Treatment strategies must be individualized so as to provide adequate local control with subsequent acceptable functional and esthetic outcome. Despite the applied therapeutic method, due to the high recurrence rate, these patients must be closely followed by clinical examination and radiographic studies.

## Abbreviations

MRI: Magnetic resonance imaging; RT: Radiation therapy.

## Consent

Written informed consent was obtained from the patient for publication of this case report and accompanying images. A copy of the written consent is available for review by the Editor-in-Chief of this journal.

## Competing interests

The authors declare that they have no competing interests.

## Authors' contributions

All authors contributed equally to this work. KI and KG, performed the surgery, PA and TS performed the pathological examination of the excised specimen, KK, CJ and KN reviewed the current literature. KG, EL and AM contributed in writing and revising the manuscript. All authors read and approved the final manuscript.
